# Resilience of Spontaneously Hypertensive Rats to Secondary Insults After Traumatic Brain Injury: Immediate Seizures, Survival, and Stress Response

**DOI:** 10.3390/ijms26020829

**Published:** 2025-01-19

**Authors:** Ilia Komoltsev, Olga Kostyunina, Pavel Kostrukov, Daria Bashkatova, Daria Shalneva, Stepan Frankevich, Olga Salyp, Natalia Shirobokova, Aleksandra Volkova, Aleksandra Soloveva, Margarita Novikova, Natalia Gulyaeva

**Affiliations:** 1 Department of Functional Biochemistry of the Nervous System, Institute of Higher Nervous Activity and Neurophysiology, Russian Academy of Sciences, Moscow 117485, Russia; komoltsev.ilia@gmail.com (I.K.);; 2 Moscow Research and Clinical Center for Neuropsychiatry, Moscow 115419, Russia

**Keywords:** traumatic brain injury; spontaneously hypertensive rats; Sprague Dawley rats; immediate seizures; mortality, corticosterone, microglia, hippocampus, dentate gyrus

## Abstract

Traumatic brain injury (TBI) is one of the primary causes of mortality and disability, with arterial blood pressure being an important factor in the clinical management of TBI. Spontaneously hypertensive rats (SHRs), widely used as a model of essential hypertension and vascular dementia, demonstrate dysfunction of the hypothalamic–pituitary–adrenal axis, which may contribute to glucocorticoid-mediated hippocampal damage. The aim of this study was to assess acute post-TBI seizures, delayed mortality, and hippocampal pathology in SHRs and normotensive Sprague Dawley rats (SDRs). Male adult SDRs and SHRs were subjected to lateral fluid-percussion injury. Immediate seizures were video recorded, corticosterone (CS) was measured in blood plasma throughout the study, and hippocampal morphology assessed 3 months post-TBI. Acute and remote survival rates were significantly higher in the SHRs compared to the SDRs (overall mortality 0% and 58%, respectively). Immediate seizure duration predicted acute but not remote mortality. TBI did not affect blood CS in the SHRs, while the CS level was transiently elevated in the SDRs, predicting remote mortality. Neuronal cell loss in the polymorph layer of ipsilateral dentate gyrus was found in both the SDRs and SHRs, while thinning of hippocampal pyramidal and granular cell layers were strain- and area-specific. No remote effects of TBI on the density of astrocytes or microglia were revealed. SHRs possess a unique resilience to TBI as compared with normotensive SDRs. SHRs show shorter immediate seizures and reduced CS response to the injury, suggesting the development of long-term adaptative mechanisms associated with chronic hypertension. Though remote post-traumatic hippocampal damage in ipsilateral dentate gyrus is obvious in both SHRs and SDRs, the data imply that physiological adaptations to high blood pressure in SHRs may be protective, preventing TBI-induced mortality but not hippocampal neurodegeneration. Understanding the mechanisms of resilience to TBI may also help improve clinical recommendations for patients with hypertension. Limitation: since more than a half of the SDRs with prolonged immediate seizures or elevated CS 3 days after TBI have died, survivorship bias might hamper correct interpretation of the data.

## 1. Introduction

Traumatic brain injury (TBI) is one of the primary causes of mortality and disability, leading to serious long-term consequences such as cognitive impairments, post-traumatic epilepsy, and depression [1]. TBI disturbs the integrity of the blood–brain barrier (BBB), and this is an important factor underlying secondary brain injury, including edema development and neurodegeneration [2], as well as hyperexcitability [3]. Arterial blood pressure is an important aspect of the clinical management of TBI. Though hypoperfusion is associated with poor TBI outcomes, there are no clear clinical recommendations on hypertension. When hypertension occurs in the setting of TBI, treatment may induce overshoot hypotension, therefore, the treatment of acute hypertension in patients with TBI is not recommended and moderate hypertensions may potentially be “conditionally protective” [4].

Spontaneously hypertensive rats (SHRs) are a unique rat strain demonstrating behavioral and physiological alterations. Spontaneous arterial hypertension in these rats is accompanied by big and small vessel angiopathy [5] and hippocampal hypoperfusion as well as by cognitive disorders [6,7]. After TBI, permeability of the BBB increased in the SHRs as compared to the normotensive rats, and this was accompanied by increased pro-inflammatory cytokine expression 2 weeks after trauma [8]. SHRs are also known to possess abnormal stress responses [9] that might affect the consequences of TBI as a stressful event. Surprisingly, the long-term effects of TBI in SHR rats have not attracted enough attention so far and remain obscure. Intuitively, being aware of multiple widely known detrimental consequences of hypertension, we might suggest that chronically elevated arterial pressure would most probably aggravate TBI. However, theoretically, SHRs may be either more vulnerable or more resistant to the acute and delayed effects of TBI because of their chronic adaptation to hypertension and altered stress reactivity.

The hippocampus, a limbic structure selectively vulnerable to stress, controlling both cognitive and affective functions, is regarded as a key brain region involved in the development of late post-traumatic pathology [10,11]. Notably, distant damage to the hippocampus after TBI in animal models shows structural similarity to damage induced by chemoconvulsants [12], indicating post-traumatic seizures as a potential mechanism mediating hippocampal damage. Excessive glutamate release induced by TBI stimulates apoptotic and necrotic cell death as a result of excitotoxicity [13,14,15]. Recently, we have studied immediate seizures and early epileptiform activity in the hippocampus of normotensive rats after TBI and revealed that the duration of acute post-traumatic seizures correlated with post-trauma corticosterone (CS) elevation and neuronal loss in the hippocampus as well as with increased mortality [16]. We have also demonstrated that the appearance of epileptiform spikes is associated with neuroinflammation and may be a hallmark of progressive neuronal loss one week after TBI in both experimental and clinical conditions [17].

A key mechanism of secondary post-TBI damage to vulnerable brain regions (in particular, the hippocampus) is neuroinflammation, a process closely related to an acute release of glucocorticoids (GCs) triggered by the physiological stress of the injury and eventually resulting in neurodegeneration [18]. GCs play a dual role in both central and peripheral inflammatory processes. On the one hand, they are believed to activate immunosuppressive mechanisms, potentially reducing acute inflammatory responses and tissue damage in the first hours after TBI. On the other hand, their prolonged elevation contributes to increased neurodegeneration, chronic inflammation, and impaired neuroplasticity in the hippocampus, leading to distant hippocampal damage and long-term consequences, including death [19]. These specific effects make GCs a crucial link in the pathogenesis of TBI. Clinical studies show that elevated cortisol levels in the acute phase following TBI correlate with injury severity and may serve as predictors of poor outcomes. For example, in patients with low Glasgow Coma Scale scores, indicative of a severe injury, cortisol levels are significantly elevated within the first 6 h post-injury, and a further increase on the third day predicts aggravation of a patient’s condition [20]. One of the possible explanations may be an association of this mechanism with the peripheral immunosuppressive action of GCs, which increases the risk of post-trauma complications [21].

The SHRs widely used as a model of essential hypertension and vascular dementia demonstrate a neurohumoral dysfunction, primarily of the hypothalamic–pituitary–adrenal system axis, which may contribute to changes in their hippocampi as well as to the development of vascular pathology and impairment of the BBB. Changes in numerous GC-dependent processes in the hippocampus, including dysfunction of steroid hormones receptors, impairments of neurotransmitter systems, development of neuroinflammation, etc., are associated with alterations of hippocampal vulnerability to stress factors [22]. The aim of this study was to assess acute seizures, GC-dependent delayed mortality, and delayed hippocampal pathology in two rat strains: normotensive Sprague Dawley rats (SDRs) and hypertensive SHRs. We also suggested that a comparison of animals adapted to life at a different blood pressure level could be useful to reveal potential adaptive mechanisms counteracting the harmful effects of TBI or processes aggravating TBI.

## 2. Results

### 2.1. Survival Rates in SHRs and SDRs

**Figure 1:**
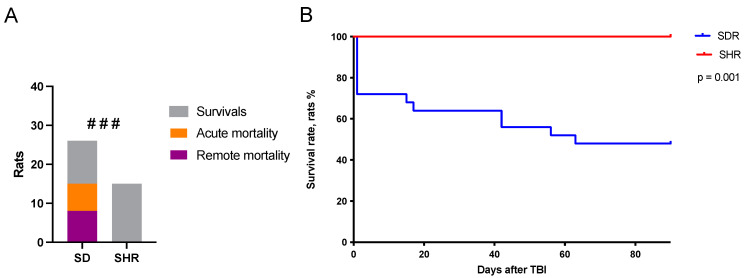
Acute and remote mortality in SDRs and SHRs. (**A**) The proportion of acute and remote mortality in SDRs and SHRs; ###—*p* < 0.001, χ^2^ test. (**B**) Survival curves according to the Kaplan–Meier method; *p* = 0.001. SHRs: TBI group n = 15 and SDRs: TBI group n = 26.

The study was conducted on male SHRs at the age when spontaneous hypertension had developed and been confirmed, as well as on normotensive SHRs of the same age. All rats from both strains underwent skull trepanation. In the TBI group, lateral fluid-percussion injury (LFPI) was performed. Sham-operated rats from both strains underwent skull trepanation without injury. The acute period (within 10 min after LFPI) was recorded on video and analyzed. The rats were then observed for three months post-surgery to assess the delayed effects of trauma. The acute survival rate (within 10 min after LFPI) and remote survival rate (within 3 months following LFPI) were significantly higher in the SHRs as compared to the SDRs (Figure 1). The overall mortality was 0% in the SHRs and 58% in the SDRs: 15 rats in total, including 7 animals (27%) in the acute phase of TBI. These results indicate higher resilience of SHRs to acute brain insults.

### 2.2. Predictors of Acute Mortality

**Figure 2:**
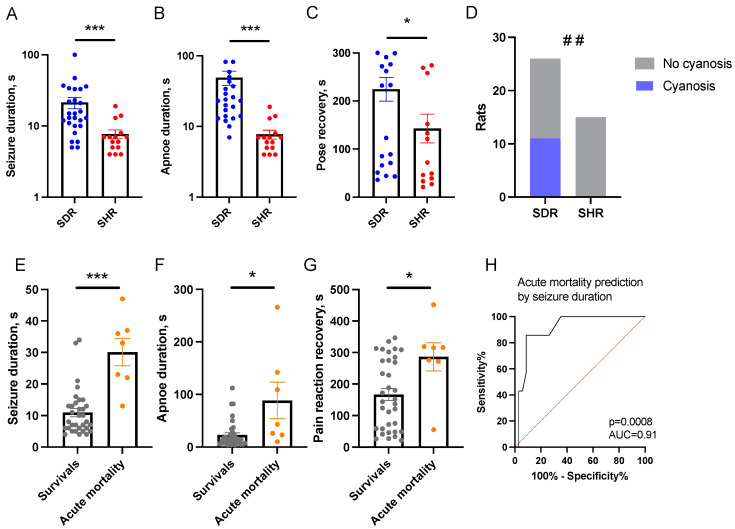
Immediate seizures in SHRs and SDRs and prediction of acute mortality. The durations of seizures (**A**), apnea (**B**), and recovery of pose (**C**) were shorter in SHRs, while rats with paw cyanosis (**D**) were absent in the SHR group. (**E**) The duration of seizures immediately after TBI was longer in the SDRs that died within 10 min after LFPI. (**F**) The duration of apnea and (**G**) recovery of pain sensitivity was longer in the SDRs that died. (**H**) ROC analysis: the duration of immediate seizures predicted acute mortality. For (**E**–**H**), mixed SDRs + SHRs group was used. (**A**–**G**)—the data are presented as M ± SEM. *—*p* < 0.05; ***—*p* < 0.001 (Mann–Whitney test); and ##—*p* < 0.01 (χ^2^ test). SHRs: TBI group n = 15 and SDRs: TBI group n = 26.

To approach the mechanisms of the SHRs’ resilience to focal brain damage, we have recorded acute seizures and changes in tail vein blood corticosterone (CS) levels on days 3 and 7, as well as at months 1 and 3, as potential predictors of mortality. The SHRs showed a significantly lower duration of immediate seizures, apnea, and pose recovery than the SDRs (Figure 2A–C). Notably, paw cyanosis, which is believed to be a marker of impaired peripheral blood circulation, was detected in 11 of the 26 SDRs and was not observed in the SHRs (Figure 2D).

Rats that died within 10 min after LFPI demonstrated more prolonged seizures (*p* < 0.001), apnea (*p* = 0.01), pain reaction recovery (*p* < 0.05) (Figure 2E–G), right and left vestibulospinal reflexes (*p* < 0.05), and a longer period of pose recovery (*p* = 0.06, statistical trend). ROC analysis showed that immediate seizure duration predicted acute mortality in the mixed SDRs + SHRs group (Figure 2H). Since mortality was observed only in the SDRs, acute seizure predictors were also valid for the SDRs alone, though with lower predictive power (*p* = 0.007, AUC = 0.85).

### 2.3. Predictors of Remote Mortality

**Figure 3:**
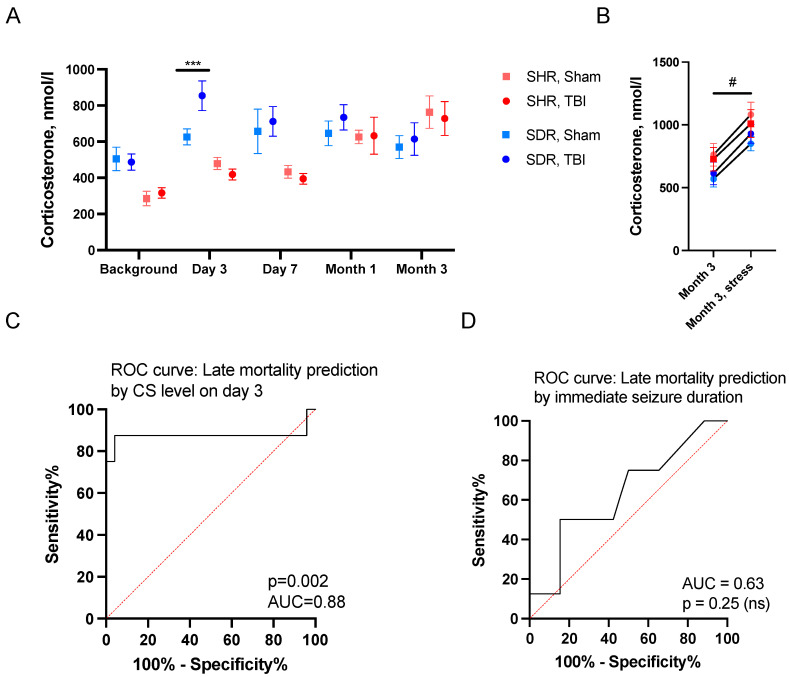
Blood serum CS time course and late mortality prediction. (**A**) Changes in CS levels during the experiment in sham-operated and TBI groups of SDRs and SHRs. An increase in CS was revealed on day 3 after TBI in SDRs. (**B**) Three months after TBI or sham operation, a short forced-swim-test-induced increase in blood plasma CS in sham-operated and TBI groups of SHRs and SDRs qq. (**C**) ROC analysis: CS level on day 3 predicted late mortality. (**D**) ROC analysis: immediate seizure duration did not predict late mortality. (**A**,**B**)—the data are presented as M ± SEM. ***—*p* < 0.005; Mann–Whitney test; #—*p* < 0.05; and RM ANOVA for time factor. For (**C**,**D**), mixed SDRs + SHRs group was used. SHRs: TBI group n = 15 and sham group n = 10 and SDRs: TBI group n = 26 and sham group n = 16.

Unlike acute mortality, the duration of immediate seizures did not predict remote mortality (Figure 3D). Previously, we have demonstrated that the level of CS in the blood increased on day 3 after LFPI in the normotensive SDRs, and this elevation predicted remote mortality [23]. Here, we aimed to explore whether this model was also valid and could predict mortality for the mixed SHRs + SDRs group.

In the SHRs, plasma CS levels increased over time (*p* < 0.001, RM ANOVA). However, TBI did not affect the CS level. In contrast, the SDRs demonstrated increased CS levels on day 3 following TBI (*p* = 0.007) (Figure 3A). Three months after craniotomy, the acute stress-challenge-induced (a short forced-swim test) CS augmentation in the blood plasma of all groups of SHRs and SDRs 30 min after the test (Figure 3B). We showed that CS levels on day 3 predicted remote mortality in the mixed SHRs + SDRs using ROC analysis (Figure 3C). CS on day 3 was also a valid predictor for the SDRs alone, though with lower predictive power (*p* = 0.01 and AUC = 0.86).

### 2.4. Effects of TBI on Hippocampal Morphology

**Figure 4:**
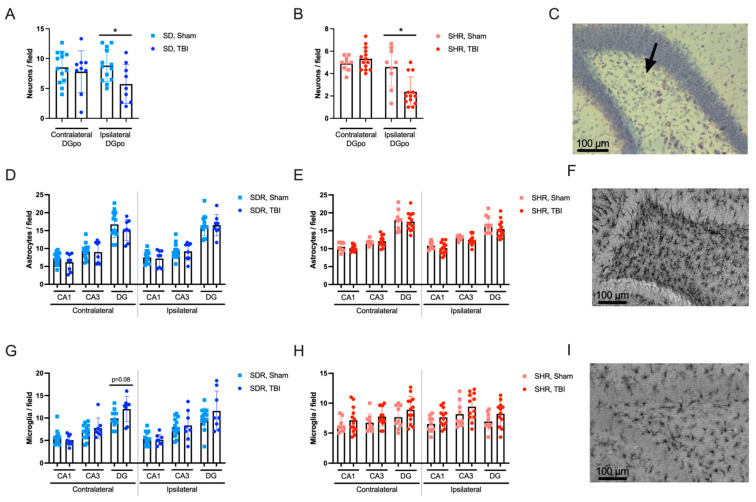
Neuronal and glial cell density in the hippocampus 3 months after TBI or sham operation. (**A**,**B**) Neuronal cell density in the DG of SDRs and SHRs, respectively. Neuronal cell density was lower in the polymorph layer of the ipsilateral hippocampus in both rat strains. (**C**) Representative microphotograph with neurodegeneration in the ipsilateral DG (polymorph layer is marked by the arrow), Nissl staining. (**D**,**E**) Astroglal cell density in the hippocampi of SDRs and SHRs, respectively. (**F**) Representative microphotograph (SHRs, ipsilateral DG), GFAP staining. (**G**,**H**) Microglial cell density in the hippocampi of SDRs and SHRs, respectively. Microglial activation was detected only in the contralateral DG of SDRs. (**I**) Representative microphotograph (SHR, ipsilateral DG), Iba1 staining. (**A**,**B**,**D**,**E**,**G**,**H**)—the data are presented as M ± SEM. *—*p* < 0.05, Mann–Whitney test. SHRs: TBI group n = 15 and sham group n = 10 and SDRs: TBI group n = 26 and sham group n = 16.

Hippocampal tissue morphology in SDRs and SHRs was studied in the DG, CA1, and CA3 subfields three months after TBI or sham operation. Neuronal cell loss in the polymorph layer of the ipsilateral DG (*p* < 0.05) was found in both the SDRs and SHRs (Figure 4A,B). Thinning of the hippocampal pyramidal cell layer was evident in the CA1 (*p* = 0.017) and CA3 (*p* = 0.068) subfields of the ipsilateral hippocampus in the SHRs and in the CA3 (*p* < 0.05) in both hemispheres of the SDRs. Thinning of the hippocampal granular cell layer was evident in the SDRs only (*p* < 0.05 for the ipsilateral and *p* = 0.075 for the contralateral hippocampus).

Unexpectedly, no differences in the density of astrocytes between sham-operated and TBI groups could be revealed in the hippocampal areas of either hemisphere in the SDRs and SHRs (Figure 4D,E, respectively). The density of microglial cells was slightly higher in the contralateral DG after TBI in the SDRs (statistical trend) (Figure 4G). No long-term effects of TBI on microglial cell density could be revealed in either hemisphere of the SHRs (Figure 4H). These data demonstrate similar morphological alterations in the hippocampi of SDRs and SHRs 3 months after TBI, though most of the SDRs with prolonged immediate seizures or elevated CS on day 3 died, possibly causing survivorship bias [23].

## 3. Discussion

In this study, we assessed both acute and long-term survival in hypertensive rats of the SHR strain, as compared to normotensive SDRs, as well as the occurrence of immediate seizures, blood plasma CS levels, and morphological changes in the hippocampi of rats 3 months after TBI. We used SDRs as a normotensive control for the SHRs. While Wistar Kyoto (WKY) rats have been widely used as the classical normotensive control for SHRs, growing evidence suggests that WKY rats may represent a model of depression rather than an adequate control [24]. Some authors consider Wistar rats as one of the most suitable normotensive controls for SHRs since they exhibit a lower incidence of spontaneous seizures than WKY rats [25]. SDRs are also commonly used as a normotensive control for SHRs [26,27,28,29,30,31,32,33]. Studies of the physiological and behavioral features of different rat strains demonstrated that exploration behavior in SHRs, according to a set of parameters, was closer to SDRs, as compared to WKY or Wistar rats [6]. Taking these results into account, we used SDRs as adequate normotensive controls to the SHRs in this study.

The main finding of our study was fairly surprising: the SHRs, despite high blood pressure, demonstrated evident resilience to TBI, with short- and long-term survival rates being significantly higher than in the normotensive SDRs. The relationships between immediate seizures, blood CS levels during the acute TBI period, and mortality (both acute and delayed) were different in the SHRs and SDRs. In the normotensive SDRs, immediate post-traumatic seizures were associated with acute mortality, whereas the SHRs demonstrated shorter seizures and showed no signs of the peripheral hemodynamic disturbances evaluated by paw cyanosis seen in 42% of the normotensive SDRs after TBI. On the third day after TBI, the CS levels in blood plasma were elevated in the normotensive SDRs, and this elevation predicted late mortality. No CS elevation could be found in the blood plasma of the SHRs. Three months after TBI in both the SHRs and the surviving SDRs, hippocampal damage was manifested as neurodegeneration (neuronal cell loss) in the ipsilateral polymorph layer of the DG. TBI-induced modest thinning of hippocampal pyramidal and granular cell layers was strain- and area-specific. TBI exerted only minor effects on astroglial and microglial cell density, which is not surprising 3 months after TBI, when acute neuroinflammatory events demonstrated in our previous study [34] have already ended. Importantly, fewer expressed immediate seizures and no mortality events in the SHRs were not accompanied by lesser neuronal cell loss in the hippocampus. We can suggest that differences between the SHRs and SDRs in their acute response to TBI and dissimilar events during the early post-trauma period underlie the resilience of the SHRs and the vulnerability of the SDRs. The long-term effects on neuronal cell loss in the polymorph layer of the ipsilateral dentate gyrus may reflect similar (for both strains) distant GC-mediated long-term effects of TBI on the hippocampus. In general, the data from this study strongly suggest that adaptation to permanently high arterial pressure in SHRs may build up the mechanisms strengthening their resilience and preventing TBI-induced mortality.

The resistance of the SHRs to TBI may be associated with the adaptation to physiological stress at the level of the cardiovascular system. As compared to normotensive rats, elevated blood pressure in SHRs is accompanied by changes in arterial stiffness [5] and endothelial dysfunction, underlying pathological alterations in arteries and arterioles [35,36,37]. Disturbances in brain microcirculation is a hallmark of hypertension, and, in SHRs, the hippocampal arterioles have a reduced diameter [38]. Importantly, hypertrophic vascular remodeling and diminished vasodilation capacity are most pronounced in the vessels of the hippocampus as compared to other regions of the brain [7]. In the SHRs, the increased permeability of the BBB, compared to that observed in the normotensive rats, induced increased pro-inflammatory cytokine expression 2 weeks after TBI [8]. However, hypertrophy and hyperplasia of smooth muscle cells in the walls of cerebral vessels, along with thickening of the meninges due to edema and excess collagen fibers, may serve as mechanical protection to the brain during TBI. In the SHRs, no signs of hemodynamic disturbances were observed in our study, which was in contrast to the SDRs. One of the reasons underlying this phenomenon may be a possibility that, in SHRs, TBI did not cause fast brain swelling or compression of vital brainstem centers, which is the most common cause of death in patients with severe TBI-related brain edema. The potential protective effects of arterial hypertension may also be explained by chronic adaptive changes in the cardiovascular system. In a seizure model induced by chemoconvulsant injection, the development of acute pulmonary hypertension within the first 5 min of seizure onset was shown to be involved in the fatal outcomes in rats, with left atrial pressure reaching over 600% of normal levels and pulmonary artery pressure over 200% [39]. Such a sharp increase in pressure in the pulmonary circulation was associated with seizure-related mortality in rats. This may suggest that cardiovascular system adaptations to extreme physiological stress may contribute to the resilience of SHRs to injury.

The resilience of the SHRs and their high long-term survival may also be associated with alterations in stress reactivity associated with changes in the hypothalamic–pituitary–adrenocortical axis. The GCs, executive hormones of this axis, are involved in neuroinflammation processes, particularly in the hippocampus, which is highly sensitive to focal brain damage [18]. GCs control stress responses, and their receptors are highly expressed in the hippocampus. The effects of GCs in the hippocampus are mediated by high-affinity mineralocorticoid receptors and low-affinity glucocorticoid receptors, which are activated by elevated levels of GCs. Two types of these receptors, cytoplasmic/nuclear receptors associated with slow genomic actions, and membrane-bound receptors mediating fast non-genomic effects of GCs, may be involved in the development of stress responses, an intricate multi-level phenomenon involving brain regions and peripheral tissues. After TBI, the accumulation of circulating GCs in the hippocampus contributes to bilateral hippocampal damage [11,40]. The effects of GCs on neuronal excitability mediated by mineralocorticoid and glucocorticoid receptors are concentration dependent: at low doses, GCs amplify glutamatergic synaptic transmission via membrane-bound mineralocorticoid receptors, while at higher doses, they decrease it via glucocorticoid receptors [41,42]. GCs regulate neuronal excitability, influence neuroinflammation, and may play a key role in hippocampal sensitivity to initial excitotoxic damage and subsequent secondary neuronal death after TBI [43]. GCs were shown to increase the vulnerability of neurons to excitotoxic damage [44], and the effect of GCs on seizures in rats is realized through the modulation of neuronal excitability [45,46]. The multiple CGs-dependent mechanisms include suppression of glucose transport, inhibition of glutamate reuptake by astrocytes, changes in Ca^2+^ concentration, and repression of neurotrophic factors production [11]. Depending on specific situations, GCs can exhibit either pro-inflammatory or anti-inflammatory properties [47]. Importantly, GCs can act as pro-inflammatory agents in the hippocampus [34,47,48] and changes in their level after focal brain injury in animals have an impact on consequences in both acute situations and over long-term periods. Previously, we have shown that the level of GCs increases on day 3 after TBI in Wistar rats, promoting bilateral hippocampal damage during the first week [16]. We did not track changes in hippocampal morphology during the acute TBI period in this study and do not know whether early bilateral neuronal cell loss preceded long-term decreases in neuronal cell density in the polymorph layer of the ipsilateral hippocampus, which was similarly expressed in the SHRs and SDRs.

Though SHRs are reported to respond to acute stress with a sharp increase in blood CS level [9], this phenomenon may depend on the specific features of the stress factor applied, since, in our study, the response to TBI was smoothed. Previously, we reported that acute post-traumatic seizures in SDRs are correlated with blood CS elevation on day 3 after TBI [25]. In this study, changes in stress reactivity or the indirect mechanisms underlying reduced immediate seizure duration may be associated with the lack of blood CS elevation at this time point in the SHRs, benefiting long-term post-traumatic survival in these rats.

Sex hormones play a critical role in secondary TBI damage as well as in the development of the hypertensive phenotype. The role of androgens in blood pressure regulation and peripheral organ damage in hypertension has been shown [49]. In TBI, sex differences have been observed in behavioral changes, neuroinflammation, neurodegeneration, and vascular damage. Furthermore, estrogen and progesterone have been explored as potential treatments in preclinical models of brain trauma [50]. Our study was conducted only on male rats due to their more stable hormonal status over time. While our results may not be directly translatable to females, we believe that males exhibit less variability, making this model more suitable for identifying and comparing the major effects in hypertensive and non-hypertensive rats, which was the main goal of our research. Additionally, male TBI patients outnumber females, highlighting sex as an often-underestimated variable in clinical studies [51]. Further investigations are crucial to advancing our understanding of sex-related effects on TBI, either with or without arterial hypertension.

The study has other limitations, some of them related to different mortality in SHRs and SDRs. Since more than a half of the SDRs died during the experiment, survivorship bias could affect the conclusions, as we have hypothesized previously [23]. The differences in mortality caused unequal groups of rats to be used in this study. Another significant limitation of the research is the rather narrow focus of the morphological study targeting the hippocampus, though other brain regions may also be involved.

## 4. Materials and Methods

All procedures performed in research involving animals were in accordance with the ethical standards of the institution and accepted experimental practice. Experiments were performed in accordance with the principles of Basel Declaration and Directive 2010/63/EU of the European Parliament and of the Council of 22 September 2010 on the protection of animals used for scientific purposes, as well as the Order of the Ministry of Health Care of the Russian Federation no. 199 n, 1 April 2016 “On approval of the rules of good laboratory practice”, and were supervised by the Ethical Committee of the Institute of Higher Nervous Activity and Neurophysiology, Russian Academy of Sciences, Moscow, Russia. The ARRIVE Guidelines (v. 2.0) were followed for data reporting.

### 4.1. Animals

The study involved male rats of two strains: 42 SDRs and 25 SHRs. The rats were 6 months old (340–350 g BW). SDRs were purchased in the “Krolinfo” Breeding Center (Moscow Region, Russia) and SHRs were purchased in Animal Breeding Facility (the Unique Research Unit Bio-Model of the Shemyakin and Ovchinnikov Institute of Bioorganic Chemistry, Russian Academy of Sciences, Moscow Region, Russia). The rats were housed 4–5 per cage made of clear polycarbonate located in the conventional vivarium under 12:12 h light/dark cycle and with food and water available ad libitum. The research protocol was approved by the Ethics Committee of the Institute of Higher Nervous Activity and Neurophysiology, Russian Academy of Sciences (protocol #10, 10 December 2012). All measures were taken to minimize animals’ suffering.

The SHRs were divided into two groups: 15 were subjected to skull trepanation with subsequent lateral fluid-percussion injury (LFPI)—TBI group, while 10 received only skull trepanation (sham operated group). The SDRs were allocated to similar groups: 26 were subjected to skull trepanation with LFPI, and 16 had skull trepanation without injury (sham operated group). The hypertensive phenotype of SHRs was confirmed (systolic and diastolic blood pressure values at the beginning of the experiment were 184 ± 3 and 139 ± 3 mmHg, respectively, M ± SEM).

### 4.2. Experimental Design

LFPI was used as a TBI model, targeting the right somatosensory cortex in SDRs and SHRs. Seizures were video recorded immediately following the injury for 5 min and analyzed later.

To assess CS levels, blood samples were collected from the tail vein 7 days before TBI, and on days 3 and 7, as well as 1 and 3 months post-TBI for both SHRs and SDRs. At 3 months post-injury, blood was collected twice—before and 30 min after the Porsolt test, which was used as an acute stress challenge. Three months post-TBI, experimental animals were sacrificed, and the brains were used for morphological study.

### 4.3. Lateral Fluid-Percussion Injury

LFPI was used as a gold standard model for TBI in rats. All rats underwent inhalation anesthesia with 2% isoflurane. Following a midline incision to expose the skull, a 3 mm wide trepanation aperture was created in the right parietal bone (AP = 3 mm and L = 3 mm), positioned directly above the sensorimotor cortex. A plastic head from a Luer-type needle was affixed to the aperture with acrylic glue to serve as a hub for pressure application from the LFPI device (Fluid Percussion Device with the PC-Based Pressure Measurement Unit, Model FP302, Richmond, VA, USA). Upon regaining full consciousness post-surgery, the animals were placed in a square Styrofoam box measuring 60 cm on each side, with 40 cm high walls, to ensure their safety and protect them from further trauma during potential seizures. LFPI was administered through the aperture, while sham-operated rats underwent all surgical procedures (anesthesia, craniotomy, and connection to the LFPI device) except for the LFPI application. The LFPI severity averaged 2.7 ± 0.1 atm (M ± SEM). After completing all procedures, the animals were returned to their home cages.

### 4.4. Immediate Seizure Visualization

Visual examination was conducted on all recorded footage to evaluate total seizure duration, tonic and clonic phase durations, incidence, and duration of jumps and apnea periods, as well as recovery times for right- and left-sided righting reflexes, posture, and pain sensitivity [16].

### 4.5. Acute Stress Challenge

To assess the impact of TBI on stress-response of SHRs and SDRs, Porsolt forced-swim test was used. The procedure was similar to that described in [52], with one modification concerning the duration of the test, which was 5 min in our study.

### 4.6. Histological Analyses

Experimental animals were sacrificed for histological analysis three months after TBI or sham surgery. The animals were euthanized under general chloral hydrate anesthesia (intraperitoneal injection, 10% solution). Arterial perfusion with a 0.9% sodium chloride solution and then 4% formaldehyde solution was performed. The brains were extracted and postfixed in a 4% formaldehyde solution. The histological studies were performed as describes previously [16,34]. In brief, brain sections of 50 μm thickness were prepared using a vibratome. Sections spaced 600 μm apart, with coordinates between 2.1 and 5.8 mm from the bregma, were selected for analysis. The Nissl method with cresyl violet staining was used. For immunohistochemical staining of the microglial marker, ionized calcium-binding adaptor molecule 1 (Iba1), floating sections were washed in phosphate-buffered saline (PBS), rinsed in PBST (0.01 M PBS with 0.3% Triton X-100), incubated in a blocking solution (5% normal goat serum in PBST), then left in rabbit anti-Iba1 antibody (FUJIFILM Wako, Osaka, Japan) solution (1:500) at 4 °C overnight. Sections were washed in PBST and incubated in goat anti-rabbit immunoglobulin G (IgG) conjugated with horseradish peroxidase antibodies (Sigma Aldrich, Darmstadt, Germany) solution (1:500) for 2 h. After that, samples were washed with PBS and incubated with SIGMAFAST 3,3′-Diaminobenzidine (DAB) peroxidase substrate with metal enhancer (Sigma Aldrich, Darmstadt, Germany) for 5 min. For immunohistochemical staining of glial fibrillary acidic protein (GFAP), a marker for astrocytes, floating brain sections were initially washed in PBS and then rinsed in PBST. The sections were incubated in a blocking solution containing 5% normal goat serum (MP Biomedicals, Irvine, CA, USA) in PBST to prevent nonspecific binding. Next, the sections were incubated overnight at 4 °C with rabbit anti-GFAP antibody (Dako, Glostrup, Denmark) solution (1:500) and goat anti-rabbit immunoglobulin G (IgG) conjugated with horseradish peroxidase antibodies (Sigma Aldrich, Darmstadt, Germany) solution (1:500) for 2 h. After that, samples were washed with PBS and incubated with SIGMAFAST 3,3′-Diaminobenzidine (DAB) peroxidase substrate with metal enhancer (Sigma Aldrich, Darmstadt, Germany) for 5 min. The samples were mounted using hard-mounting medium.

Microphotographs of Nissl-stained and immunohistochemically stained sections were taken using a Keyence BZ-X700 microscope (Keyence Corporation of America, Itasca, IL, USA. The density of microglial, astroglial, and neuronal cells was calculated as the number of cells in a 150 × 150 μm visual field in the polymorph layers of the dentate gyrus (DG), as well as CA1 and CA3 subfields of the hippocampus. ImageJ 1.52q program was used for cell count.

### 4.7. Measurement of CS in Blood Plasma

Blood samples were collected in Eppendorf tubes with 10 µL of heparin and centrifuged at 1500× *g* at 4 °C for 15 min to obtain plasma. CS levels in blood plasma were measured using enzyme-linked immunosorbent assay (ELISA). ELISA kits were used to measure both free and bound CS levels in blood plasma (CS ELISA, DRG, Marburg, Germany). Aliquots of plasma were stored at −80 °C before measurements.

### 4.8. Statistical Analysis

Statistical analysis was performed using jamovi 2.6.2 (Sydney, Australia); visualizations were created in GraphPad Prism 8 (Boston, MA, USA). Survival rates between SDRs and SHRs were compared using the log-rank test and Kaplan–Meier method. Seizure parameters did not correspond to normal distribution and were analyzed using chi-square test and Mann–Whitney U-test. CS level dynamics were assessed using repeated measures (RM) ANOVA. Receiver operating characteristic (ROC) analysis and area under curve (AUC) were applied to develop predictive models for early and late mortality. The Mann–Whitney U-test was used to compare independent variables (sham vs. TBI groups), the Wilcoxon test was used for comparison of dependent variables (ipsilateral vs. contralateral hemispheres). Data are presented as mean ± standard error of mean (M ± SEM).

## 5. Conclusions

This study demonstrates that hypertensive SHRs show a unique resilience to TBI, with both high acute and long-term survival rates, as compared with normotensive SDRs. SHRs exhibit shorter immediate seizures, no acute post-trauma hemodynamic alterations, and reduced acute CS response to injury, suggesting a protective adaptation, which is presumably associated with chronic hypertension. However, three months post-TBI, hippocampal damage and ipsilateral neurodegeneration in the DG was evident in both SHRs and surviving SDRs. These findings suggest that physiological adaptations to high blood pressure in SHRs may be protective, preventing TBI-induced mortality but not hippocampal neurodegeneration.

## Data Availability

The datasets used and/or analyzed during the current study are available from the corresponding author on reasonable request.

## Abbreviations

AUC	area under the receiver operating characteristic (ROC) curve
BBB	blood–brain barrier
CS	corticosterone
DG	dentate gyrus
ELISA	enzyme-linked immunosorbent assay
GCs	glucocorticoids
GFAP	glial fibrillary acidic protein
Iba1	ionized calcium-binding adaptor molecule 1
IgG	immunoglobulin G
LFPI	lateral fluid-percussion injury
PBS	phosphate-buffered saline
PBST	PBS Triton X-100 solution
ROC	receiver operating characteristic
SHRs	spontaneously hypertensive rats
SDRs	Sprague Dawley rats
TBI	traumatic brain injury
WKY	Wistar Kyoto
